# Perinatal and postnatal exposures and risk of young-onset breast cancer

**DOI:** 10.1186/s13058-020-01317-3

**Published:** 2020-08-13

**Authors:** Mary V. Diaz-Santana, Katie M. O’Brien, Aimee A. D’Aloisio, Gloria Regalado, Dale P. Sandler, Clarice R. Weinberg

**Affiliations:** 1 Biostatistics and Computational Biology Branch, National Institute of Environmental Health Sciences, 111 TW Alexander Dr., Research Triangle Park, Durham, NC 27709 USA; 2 Epidemiology Branch, National Institute of Environmental Health Sciences, 111 TW Alexander Dr., Research Triangle Park, Durham, NC 27709 USA; 3 Social and Scientific Systems, Inc., 4505 Emperor Blvd, Suite 400, Durham, NC 27703 USA; 4 Institute for Advanced Analytics, North Carolina State University, 901 Main Campus Drive, Suite 230, Raleigh, NC 27606 USA

**Keywords:** Perinatal exposures, Postnatal exposures, Young-onset breast cancer, Hormone receptor status, Birth weight, Preeclampsia, Breastfeeding

## Abstract

**Background:**

*Perinatal* factors have been associated with some adult health outcomes, but have not been well studied in young-onset breast cancer. We aimed to evaluate the association between young-onset breast cancer and perinatal exposures and to explore etiologic heterogeneity in the relationship between associated perinatal factors and estrogen receptor status of the tumor.

**Methods:**

We addressed this in a sister-matched case-control study. Cases were women who had been diagnosed with ductal carcinoma in situ or invasive breast cancer before the age of 50. Each case had a sister control who was free of breast cancer up to the same age at which her case sister developed the disease. The factors considered were self-reported and included the mother’s preeclampsia in that pregnancy, mother’s smoking in that pregnancy, gestational hypertension, prenatal diethylstilbestrol use, and gestational diabetes, as well as low birth weight (less than 5.5 pounds), high birth weight (greater than 8.8 pounds), short gestational length (less than 38 completed weeks), and being breastfed or being fed soy formula.

**Results:**

In conditional logistic regression analyses, high birth weight (odds ratio [OR] = 1.59, 95% confidence interval [CI] 1.07–2.36) and preeclampsia (adjusted OR = 1.92, CI 0.824–4.5162) were positively associated with risk. The association with preeclampsia was stronger when the analysis was restricted to invasive breast cancer (OR = 2.87, CI 1.08–7.59). We also used case-only analyses to assess etiologic heterogeneity for estrogen receptor (ER)-positive versus estrogen receptor-negative cancer. Women who were born to a preeclamptic pregnancy and later developed young-onset breast cancer were at increased odds for the ER-negative type (OR = 2.27; CI 1.05–4.92).

**Conclusion:**

These results suggest that being born to a preeclamptic pregnancy may increase risk for young-onset breast cancer, especially for the ER-negative subtype.

## Background

Breast cancer is common in the United States (US), with approximately one in eight women diagnosed during their lifetime [1]. Incidence increases with age, but roughly 18% occur before the age of 50 years [1]. Breast cancer risk factors have been reported to vary across age of onset [2, 3], which suggests that there is etiologic heterogeneity of breast cancer associated with age at onset (young-onset vs. older-onset).

According to a provocative hypothesis proposed by Trichopoulos in 1990, some breast cancers may etiologically originate in utero [4]*.* He proposed that prenatal exposure to endogenous or exogenous hormones might influence the trajectory of breast cancer development later in life, implying that a critical window for carcinogenesis of the breast might begin during prenatal life. For example, intrauterine exposure to insulin-like growth factors has been proposed to have a role in breast cancer risk [5]. Evaluating the effects of very early life exposures poses methodological challenges, since few investigators have had data to characterize the intrauterine environment and relate that to a subsequent breast cancer diagnosis. However, some epidemiological studies have examined this hypothesis by using surrogate markers for in utero exposures to steroids and growth hormones.

One example of this is preeclampsia, which may be associated with high maternal concentrations of androgens and low concentrations of estrogen [6, 7]. Because preeclampsia is also a complication that may require early termination of the pregnancy [8], daughters born from a preeclamptic pregnancy may, on average, have had hormonally different gestational environments due both to lower levels and to shorter gestations. A meta-analysis [9] assessing the role of maternal preeclampsia on the offspring’s later risk of breast cancer found a significant inverse association (risk ratio (RR) 0.48; 95% CI 0.30–0.78) [9]. However, results from studies [10–14] assessing the impact of maternal preeclampsia on daughters’ risk specifically for young-onset breast cancer have been inconsistent.

Other perinatal factors, such as birth weight and birth length, might reflect in utero exposure to growth hormones [15]. Most of the research studies evaluating the association between birth weight and risk among young women have reported a modest increase in breast cancer risk associated with high birth weight [14, 16–23]. Another perinatal factor that may influence a woman’s subsequent risk of breast cancer is exposure to breast milk [24]. A meta-analysis undertaken to assess the relationship between breastfeeding during infancy and adult breast cancer incidence found no overall relationship (RR 0.94; 95% CI 0.85–1.04) but found evidence for a reduced risk of premenopausal breast cancer (RR 0.88; 95% CI 0.79–0.98) [25]. Other feeding modalities in infancy have been evaluated to gain a better understanding of their impact on breast cancer risk. Soy contains phyto-estrogens, and a population-based case-control study [26] assessed the association between soy formula consumption during infancy and breast cancer and reported that being fed only soy formula during the first 4 months of age was not associated with an increased odds of having developed breast cancer (OR 0.42; 95% CI 0.13–1.40). Other perinatal factors that have been previously studied as potential risk factors for breast cancer risk include twin membership, parental age at delivery, and gestational length [13, 14, 27].

Evaluating the role of rare perinatal factors such as preeclampsia and twin membership on young-onset breast cancer requires a large sample of women. Many of the previous studies of perinatal factors and breast cancer risk have used registry data [11, 12, 14, 27, 28] while others have been hampered by small numbers of breast cancer cases. While using registry data can provide a large sample size and reduces the influence of recall and selection bias, data on some perinatal factors may not be available. In addition, confounder adjustment in the analysis is necessarily limited to variables that were collected in the registry’s database. We conducted a sister-matched case-control analysis of participants in the Two Sister Study to assess the effect of perinatal factors on young-onset (under age 50) breast cancer risk. The sister-matched case-control design provides a unique opportunity to partially control for possible confounding factors not measured in the study that could tend to be shared among sisters, such as their mother’s body mass index (BMI).

## Methods

### Study population and design

Participants in The Two Sister Study were enrolled between 2008 and 2010. Participants were eligible as cases if they had been diagnosed with breast cancer before 50 years of age and within the previous 4 years and had one or more unaffected sisters who had enrolled in the Sister Study (2003–2009). Sister Study participants had to be 35–74 years of age at the time of enrollment and had to live in the United States or Puerto Rico. Participants in the Sister Study had reported on their sister’s earlier breast cancer diagnosis at the time of their enrollment, and the list of families to target for recruitment into the Two Sister Study was based on that information. The sisters with a previous breast cancer diagnosis who appeared to be eligible were contacted through their participating sister, who was asked to send their affected sister a letter inviting their participation. Case sisters who then contacted the study and were found to be eligible were administered the same computer-assisted telephone interviews used in the Sister Study and another questionnaire about their breast cancer diagnosis. Their consent was requested for retrieval of their breast cancer medical record, which was used to collect pathological data on tumor characteristics (including estrogen receptor (ER) status). When medical records were not provided, we relied on self-report. We were able to obtain medical records or pathology reports to confirm the diagnosis for 88.7% breast cancer cases, and we relied on self-report for the remaining 11.3%. Very high positive predictive values for self-reported breast cancer (> 99.0%) have been reported for incident breast cancer cases in the Sister Study [29].

Because of the sampling design, controls were often older than their case sister. To address this potential source of bias, we restricted our controls to participants who had remained disease-free (breast cancer) up to the age at which their case sister was diagnosed with breast cancer, so that either could have been the case. The sample for the Two Sister Study was augmented by recruiting additional incident young-onset cases that were diagnosed during follow-up of the Sister Study cohort through September 2016, netting an additional 243 cases, many of whom also had an unaffected Sister Study sister who was also being followed. Those that did not have a control sister were nonetheless included in the case-only analyses to study etiologic heterogeneity. In total, 1759 cases and 1672 sister controls (including the total numbers to be included in matched analyses or case-only analysis) were enrolled in the Two Sister Study.

### Exposures of interest

Perinatal factors obtained via questionnaire at study enrollment included participant’s birth weight, used to identify those with high birth weight (≥ 8.8 pounds) or low birth weight (≤ 5.5 pounds); participant’s gestational age at birth, used to categorize them as less than 38 weeks or not; and whether their gestation was complicated by diethylstilbestrol exposure, preeclampsia, eclampsia, gestational hypertension, or gestational diabetes. We dichotomized gestational age into < 38 weeks or ≥ 38 weeks because the question women had been asked was as follows: Were you born within 1 week of your mother’s due date, more than 1 week before her due date, or more than 1 week after her due date? If the participant reported that she was born early or late, she was further asked the number of weeks (less than 2 weeks, 2–4 weeks, 1–2 months, more than 2 months, I do not know). We also ascertained some postnatal exposures: having been breastfed as an infant and history of soy formula consumption during infancy.

Data on perinatal and postnatal exposures were from a “Family History” questionnaire mailed to all participants at enrollment. Participants were encouraged to speak to their mothers (or other family members) to improve accuracy and most questions allowed qualified answers (e.g., options of definitely yes, probably yes, probably no, definitely no). In addition, we carried out a validation study (manuscript in preparation) in which mothers of participants who were under 60 at enrollment were asked to complete the same questionnaire. Agreement was good for most variables, with kappas ranging from 0.5 to 0.9, but mother’s data was also retrospectively reported. Examiners retrieved these questionnaires from Sister Study participants when conducting home visits for blood collection. Because most cases were identified retrospectively and had no home visit, they had to mail in their questionnaire. The cases consequently had more missing data than controls.

### Covariate data

Data on participants’ demographics (e.g., age, race/ethnicity) and height and weight (used to calculate body mass index) were obtained at enrollment.

#### Statistical analysis

We provide descriptive statistics for cases and controls separately for each pre- and perinatal factor. Since there was a small fraction of data missing for some of the covariates and exposures of interest, we conducted a complete case analysis [30]. We used conditional logistic regression to calculate odds ratios (OR) and 95% confidence intervals (CI) to explore the association between perinatal factors and young-onset breast cancer. Each pre- and perinatal factor was considered as the primary exposure variable in a separate regression model. Variables of known biological importance in the perinatal factors-breast cancer relationship were evaluated for inclusion as potential effect modifiers (e.g., maternal smoking while pregnant with the study participant). In addition, we used directed acyclic graphs to identify potential confounders. For the analyses of preeclampsia, gestational hypertension, and their composite, i.e., “any” hypertensive disorder, we adjusted for whether or not the participant was their mother’s first baby and whether or not the mother had smoked in that pregnancy. For analyses of low and high birth weight and short gestational length, we adjusted for preeclampsia and for maternal smoking in that pregnancy. In a further sensitivity analysis, we tried to isolate the effect of gestational preeclampsia by restricting the preeclampsia analysis to those participants who had not themselves had a preeclamptic pregnancy. A total of 341 women (155 cases and 186 controls) had a preeclamptic pregnancy themselves and were excluded from this analysis. The reasoning for that approach was that participants from a preeclamptic pregnancy may have inherited a propensity to develop this complication in their own pregnancies, and the experience of having a preeclamptic pregnancy may confer reduced breast cancer risk [31]. We repeated these analyses with restriction to invasive cases and their sisters (*N* = 2717, 1250 cases and 1467 controls) and stratifying by ER status.

We also conducted case-only analysis to explore etiologic heterogeneity in the relationship between perinatal factors and the ER status of the breast tumor [32]. The case-only analysis used unconditional logistic regression, treating ER− breast cancer as the outcome. We also carried out a sensitivity analysis where we restricted to invasive cases. All the statistical analyses were conducted using STATA version 15.1 (Stata Corp, College Station, TX) and SAS version 9.4 (SAS Institute, Cary, NC).

## Results

Table 1 shows selected characteristics of cases and controls. Control sisters were slightly older than their case sisters (46.3 vs. 45.0 years old at the time of the case sister’s diagnosis). The percentages of cases born with a low birth weight (8.4%) or a high birth weight (9.4%) were slightly higher than those for control sisters (6.7% low birth weight and 7.3% high birth weight). More cases than controls were born with a gestation less than 38 weeks (15.2 vs. 11.5%).

**Table 1:** Baseline characteristics and in-utero exposures by case status (*N* = 3431)

Characteristics mean ± SD	Controls (*n* = 1672)	Cases (*n* = 1759)
**Age at the time of case’s diagnosis**	46.3 ± 6.0	45.0 ± 3.9
**Body mass index**	23.8 ± 4.4	23.8 ± 4.2
Characteristics, *n* (%)	*n* (%)	*n* (%)
**Age at the time of case’s diagnosis**		
< 40	272 (16.3)	211 (12.0)
40–44	406 (24.3)	491 (27.9)
≥ 45	994 (59.5)	1057 (60.1)
**Race**		
Non-Hispanic white	1489 (89.1)	1520 (86.4)
Non-Hispanic black	73 (4.4)	107 (6.1)
Hispanic	66 (3.9)	79 (4.5)
Other	43 (2.6)	53 (3.0)
**Education**		
High school or less	206 (12.3)	214 (12.2)
Some college but no degree	273 (16.3)	263 (15.0)
Associate or technical degree	260 (15.6)	249 (14.1)
Bachelor degree	530 (31.7)	589 (33.5)
Master or doctoral degree	403 (24.1)	444 (25.2)
**BMI**		
< 24.9	1216 (72.9)	1257 (71.7)
25–29.9	306 (18.4)	362 (20.7)
≥ 30	146 (8.8)	134 (7.6)
**Birth order**		
First (oldest)	956 (57.2)	829 (47.1)
Second	580 (34.7)	871 (49.5)
Third or younger	136 (8.1)	59 (3.4)
**Mother’s smoking history while pregnant with participant**		
No	1117 (69.5)	1024 (69.2)
Yes	491 (30.5)	455 (30.8)
**Mother’s gestational diabetes**		
No	1561 (99.1)	1428 (99.2)
Yes	14 (0.9)	11 (0.8)
**Mother’s preeclampsia/ eclampsia or toxemia**		
No	1507 (98.2)	1359 (97.7)
Yes	27 (1.8)	32 (2.3)
**Mother’s any hypertensive disorder**		
No	1506 (96.2)	1368 (95.7)
Yes	60 (3.8)	62 (4.3)
**Mother’s pregnancy-induced hypertension**		
No	1445 (97.0)	1282 (96.7)
Yes	44 (3.0)	44 (3.3)
**Was this participant breastfed?**		
No	948 (59.6)	948 (64.3)
Yes	642 (40.4)	527 (35.7)
**Participant’s soy formula feeding history**		
No	1415 (95.8)	1221 (95.5)
Yes	62 (4.2)	58 (4.5)
** Participant’s in-utero DES exposure**		
No	1432 (96.8)	1252 (97.1)
Yes	48 (3.2)	38 (2.9)
** Low birth weight**		
Birth weight > 5.5 pounds	1475 (93.3)	1368 (91.6)
Birth weight ≤ 5.5 pounds	106 (6.7)	125 (8.4)
**High birth weight**		
Birth weight < 8.8 pounds	1257 (92.7)	1114 (90.6)
Birth weight ≥ 8.8 pounds	99 (7.3)	115 (9.4)
**Short gestational length at birth**		
Born ≥ 38 weeks	988 (88.5)	769 (84.8)
Born < 38 weeks	128 (11.5)	138 (15.2)

Following variables had missing data: BMI (5 controls and 1 cases), race (1 controls and 0 cases), maternal smoking during the participant’s gestation (64 controls, 280 cases), maternal gestational diabetes during the participant’s gestation (97 controls and 320 cases), maternal history of preeclampsia/eclampsia or toxemia during the participant’s gestation (138 controls and 368 cases), maternal hypertensive disorder during the participant’s gestation (106 controls and 329 cases), maternal pregnancy-related hypertension during the participant’s gestation (controls 183 and cases 433), whether the participant was breastfed (controls 82 and cases 284), participant’s soy formula feeding history (controls 195 and cases 480), mother’s DES exposure during the participant’s gestation (controls 192 cases 469), low birth weight (controls 91 and cases 266), high birth weight (controls 316 and cases 530), and short gestational age at birth (controls 603 and cases 893)

In general, we observed weak associations between perinatal factors and breast cancer (Table 2). We excluded gestational diabetes from consideration because few women reported it. There was a positive association between breast cancer and high birth weight (OR = 1.59, 95% CI 1.07–2.36). Participants who had a short gestational length (< 38 weeks) (OR = 1.26, 95% CI 0.83–1.92) and those whose mother smoked during their gestation (OR = 1.19, 95% CI 0.81–1.74) had estimated positive associations with breast cancer. After adjusting for possible confounding by whether they were their mother’s first child and maternal smoking during that pregnancy, we observed a positive association between being born to a pregnancy with pre-eclampsia and breast cancer risk (OR = 1.92, 95% CI 0.82–4.51). Further excluding from this analysis participants who themselves had had a preeclamptic pregnancy, led to a slightly increased estimated odds of breast cancer (OR = 2.28, 95% CI 0.89–5.82). Prenatal exposure to DES during pregnancy (OR = 0.82, 95% CI 0.44–1.54) and having been breastfed (OR = 0.76, 95% CI 0.58–1.00) were associated with a slightly reduced odds of breast cancer. Neither having being fed only soy formula during infancy nor having a low birth weight was associated with breast cancer (OR = 1.04, 95% CI 0.66–1.65; OR = 1.05, 95% CI 0.70–1.59, respectively). In a sensitivity analysis that excluded the sibships with in situ breast cancer, similar associations were observed with most perinatal factors. However, the point estimates associated with being born to a pregnancy with pre-eclampsia, high birth weight, and any hypertensive disorder increased (Table 3).

**Table 2:** Breast cancer odds ratio (ORs) in relation to participant’s perinatal exposures

Characteristics	Controls (***n*** = 1672)	Cases (***n*** = 1473)	OR (95% CI)	OR (95% CI)
**Preeclampsia/eclampsia/toxemia**				
No	1163 (98.4)	1026 (97.9)	1.00	1.00
Yes	19 (1.6)	22 (2.1)	1.92 (0.82–4.51)^¥^	2.28 (0.89–5.82)^€^
**Gestational hypertension**^**§**^				
No	1121 (97.6)	968 (97.9)	1.00	1.00
Yes	28 (2.4)	21 (2.1)	0.84 (0.44–1.59)^¥^	1.00 (0.50–2.00)^€^
**Any hypertensive disorder**				
No	1158 (96.1)	1029 (96.0)	1.00	1.00
Yes	47 (3.9)	43 (4.0)	1.12 (0.66–1.91)^¥^	1.40 (0.78–2.52)^€^
**Maternal smoking**				
No	935 (70.6)	821 (70.0)	1.00	
Yes	389 (29.4)	352 (30.0)	1.19 (0.81–1.74)*	
**Low birth weight**				
Birth weight > 5.5 pounds	1226 (93.2)	1082 (92.2)	1.00	1.00
Birth weight ≤ 5.5 pounds	90 (6.8)	91 (7.8)	1.14 (0.80–1.62)	1.05 (0.70–1.59)^Ŧ^
**High birth weight**				
Birth weight < 8.8 pounds	881 (92.3)	771 (90.2)	1.00	1.00
Birth weight ≥ 8.8 pounds	74 (7.87	84 (9.8)	1.59 (1.07–2.36)	1.44 (0.93–2.22)^Ŧ^
**Breastfed**				
No	780 (59.6)	726 (62.4)	1.00	
Yes	528 (40.4)	437 (37.6)	0.76 (0.58–1.00)	
**Soy-fed (formula made from soy)**				
No	1021 (95.9)	898 (95.4)	1.00	
Yes	44 (4.1)	43 (4.6)	1.04 (0.66–1.65)	
**DES exposure during pregnancy**				
No	1042 (97.0)	933 (97.4)	1.00	
Yes	32 (3.0)	25 (2.6)	0.82 (0.44–1.54)	
**Short gestational length at birth**				
Born ≥ 38 weeks	561 (88.1)	490 (85.1)	1.00	1.00
Born < 38 weeks	76 (11.9)	86 (14.9)	1.36 (0.91–2.02)	1.26 (0.83–1.92)^Ŧ^

Counts including only participants with complete information on exposure of interest

^¥^Adjusted for whether or not the participant was their mother’s first baby and for maternal smoking in that pregnancy

^§^Without preeclampsia/eclampsia

*Adjusted by birth order

^€^Adjusted for whether or not the participant was their mother’s first baby, for maternal smoking in that pregnancy and excluding women who themselves had a preeclamptic pregnancy

^Ŧ^Adjusted for mother’s preeclampsia and smoking during the participant’s gestation

**Table 3:** Breast cancer odds ratio (ORs) in relation to participant’s perinatal exposures, excluding in situ cases

Characteristics	Controls (***n*** = 1467)	Cases (***n*** = 1250)	OR (95% CI)	OR (95% CI)
**Preeclampsia/eclampsia/toxemia**	***n*** **(%)**	***n*** **(%)**		
No	990 (98.5)	867 (97.6)	1.00	1.00
Yes	15 (1.5)	21 (2.4)	2.87 (1.08–7.59)^¥^	4.03 (1.29–12.6)^€^
**Gestational hypertension**^**§**^				
No	958 (97.5)	818 (97.7)	1.00	1.00
Yes	25 (2.5)	19 (2.3)	0.91 (0.46–1.80)	1.00 (0.49–2.04)^€^
**Any hypertensive disorder**				
No	984 (96.1)	867 (98.6)	1.00	1.00
Yes	40 (3.9)	40 (4.4)	1.32 (0.75–2.32)^¥^	1.58 (0.85–2.92)^€^
**Maternal smoking**				
No	787 (70.5)	692 (70.3)	1.00	
Yes	329 (29.5)	292 (29.7)	1.13 (0.75–1.70)*	
**Low birth weight**				
Birth weight > 5.5 pounds	1033 (93.3)	907 (92.3)	1.00	1.00
Birth weight ≤5.5 pounds	74 (6.7)	76 (7.7)	1.17 (0.78–1.74)	1.14 (0.71–1.81)^Ŧ^
**High birth weight**				
Birth weight < 8.8 pounds	741 (91.8)	642 (89.4)	1.00	1.00
Birth weight ≥ 8.8 pounds	66 (8.2)	76 (10.6)	1.67 (1.10–2.54)	1.52 (0.96–2.40)^Ŧ^
**Breastfed**				
No	664 (60.2)	611 (62.7)	1.00	
Yes	438 (39.8)	364 (37.3)	0.80 (0.60–1.07)	
**Soy-fed (formula made from soy)**				
No	847 (95.6)	743 (95.5)	1.00	
Yes	39 (4.4)	35 (4.5)	0.92 (0.56–1.52)	
**DES exposure during pregnancy**				
No	872 (96.8)	776 (97.1)	1.00	
Yes	29 (3.2)	23 (2.9)	0.85 (0.44–1.65)	
**Short gestational length at birth**				
Born ≥ 38 weeks	474 (87.8)	412 (84.6)	1.00	1.00
Born < 38 weeks	66 (12.2)	75 (15.4)	1.38 (0.90–2.11)	1.28 (0.81–2.02)^Ŧ^

Counts including only participants with complete information on exposure of interest

^¥^Adjusted for whether or not the participant was their mother’s first baby and for maternal smoking in that pregnancy

^§^Without preeclampsia/eclampsia

*Adjusted by birth order

^€^Adjusted for whether or not the participant was their mother’s first baby, for maternal smoking in that pregnancy and excluding women who themselves had a preeclamptic pregnancy

^Ŧ^Adjusted for mother’s preeclampsia and smoking during the participant’s gestation

Table 4 displays results from case-only analyses. We observed that cases born to a mother with pre-eclampsia in that pregnancy (OR = 2.23, 95% CI 0.94–5.30) were at increased odds for ER-negative tumors. Additionally, cases who had been born with a high birth weight (OR = 0.64, 95% CI: 0.35–1.17) were at somewhat reduced odds of having an ER-negative tumor. We observed a near-null association between ER-negative tumors and prenatal exposure to DES (OR = 0.91, 95% CI: 0.38–2.21) and maternal smoking (OR = 1.08, 95% CI 0.81–1.45). We observed similar associations when restricting to participants with invasive cancer (Table 5). Stratified analysis by ER status (Table 6) showed that the overall association appears to be largely driven by ER-negative cancers. In addition, the overall positive association between breast cancer and high birth weight persisted among ER-positive case subjects (OR = 1.68, 95% CI 1.07–2.62).

**Table 4:** Case-only odds ratios for estrogen receptor-negative breast cancer in relation to participant’s exposures

Characteristics	ER+ (***n*** = 1356)	ER− (***n*** = 338)	OR (95% CI^**)¥**^	OR (95% CI)
**Preeclampsia/eclampsia/toxemia**	***n*** **(%)**	***n*** **(%)**		
No	1078 (98.2)	237 (95.9)	1.00	1.00
Yes	20 (1.8)	10 (4.1)	2.27 (1.05–4.92)	2.23 (0.94–5.30)*
**Gestational hypertension**^**§**^				
No	1031 (97.7)	222 (97.4)	1.00	1.00
Yes	24 (2.3)	6 (2.6)	1.16 (0.47–2.87)	1.08 (0.40–2.88)*
**Any hypertensive disorder**				
No	1084 (96.1)	237 (93.7)	1.00	1.00
Yes	44 (3.9)	16 (6.3)	1.66 (0.92–3.00)	1.56 (0.82–2.98)*
**Maternal smoking**				
No	812 (69.6)	175 (67.8)	1.00	
Yes	355 (30.4)	83 (32.2)	1.08 (0.81–1.45)	
**Low birth weight**				
Birth weight > 5.5 pounds	1075 (91.6)	247 (92.2)	1.00	1.00
Birth weight ≤ 5.5 pounds	98 (8.4)	21 (7.8)	0.93 (0.57–1.52)	0.86 (0.49–1.49)^#^
**High birth weight**				
Birth weight < 8.8 pounds	873 (90.3)	205 (92.8)	1.00	1.00
Birth weight ≥ 8.8 pounds	94 (9.7)	16 (7.2)	0.72 (0.42–1.26)	0.64 (0.35–1.17)^#^
**Breastfed**				
No	737 (63.2)	172 (67.2)	1.00	
Yes	428 (36.8)	84 (32.8)	0.84 (0.63–1.12)	
**Soy-fed (formula made from soy)**				
No	958 (95.3)	218 (96.0)	1.00	
Yes	47 (4.7)	9 (4.0)	0.84 (0.41–1.74)	
**DES exposure during pregnancy**				
No	994 (97.0)	211 (97.2)	1.00	
Yes	31 (3.0)	6 (2.8)	0.91 (0.38–2.21)	
**Short gestational length at birth**				
Born ≥ 38 weeks	617 (85.3)	131 (84.0)	1.00	1.00
Born < 38 weeks	106 (14.6)	25 (16.0)	1.11 (0.69–1.79)	0.94 (0.56–1.57)^#^

^¥^Uadjusted analysis

^§^Excluding preeclampsia/eclampsia

*Adjusted for whether or not the participant was their mother’s first baby and excluding women who themselves had a preeclamptic pregnancy

^#^Adjusted for whether the participant’s gestation was complicated by preeclampsia

**Table 5:** Case-only odds ratios for estrogen receptor-negative breast cancer (excluding in situ) in relation to participant’s exposures

Characteristics	ER+ (***n*** = 1124)	ER− (***n*** = 307)	OR (95% CI^**)¥**^	OR (95% CI)
**Preeclampsia/eclampsia/toxemia**	***n*** **(%)**	***n*** **(%)**		
No	890 (98.0)	213 (95.9)	1.00	1.00
Yes	18 (2.0)	9 (4.1)	2.09 (0.93–4.72)	2.04 (0.81–5.13)*
**Gestational hypertension**^**§**^				
No	853 (97.9)	198 (97.1)	1.00	1.00
Yes	18 (2.1)	6 (2.9)	1.44 (0.56–3.66)	1.29 (0.47–3.53)*
**Any hypertensive disorder**				
No	895 (96.1)	213 (93.4)	1.00	1.00
Yes	36 (3.9)	15 (6.6)	1.75 (0.94–3.26)	1.62 (0.81–3.20)*
**Maternal smoking**				
No	677 (70.2)	156 (67.2)	1.00	
Yes	287 (29.8)	76 (32.8)	1.15 (0.85–1.56)	
**Low birth weight**				
Birth weight > 5.5 pounds	885 (91.3)	225 (92.2)	1.00	1.00
Birth weight ≤ 5.5 pounds	84 (8.7)	19 (7.8)	0.89 (0.53–1.49)	0.80 (0.44–1.44)^#^
**High birth weight**				
Birth weight < 8.8 pounds	715 (89.4)	185 (92.0)	1.00	1.00
Birth weight ≥ 8.8 pounds	85 (10.6)	16 (8.0)	0.73 (0.42–1.27)	0.66 (0.36–1.22)^#^
**Breastfed**				
No	606 (62.9)	151 (65.4)	1.00	
Yes	358 (37.1)	80 (34.6)	0.90 (0.66–1.21)	
**Soy-fed (formula made from soy)**				
No	787 (95.5)	197 (96.1)	1.00	
Yes	37 (4.5)	8 (3.9)	0.86 (0.40–1.88)	
**DES exposure during pregnancy**				
No	818 (96.7)	189 (97.4)	1.00	
Yes	28 (3.3)	5 (2.6)	0.77 (0.29–2.03)	
**Short gestational length at birth**				
Born ≥ 38 weeks	517 (85.6)	118 (84.3)	1.00	1.00
Born < 38 weeks	87 (14.4)	22 (15.7)	1.11 (0.67–1.84)	0.91 (0.52–1.60)^#^

^¥^Uadjusted analysis

^§^Excluding preeclampsia/eclampsia

*Adjusted for whether or not the participant was their mother’s first baby and excluding women who themselves had a preeclamptic pregnancy

^#^Adjusted for whether the participant’s gestation was complicated by preeclampsia

**Table 6:** Breast cancer odds ratio (ORs) in relation to participant’s in utero exposures by ER status, with counts including only participants with complete information on exposure of interest

Characteristics	ER+ cases (1161 case subjects, 1321 control subjects)				ER− cases (290 case subjects, 328 control subjects)			
	Controls (***n*** = 1321)	Cases (***n*** = 1161)	OR (95% CI)	OR (95% CI)	Controls (***n*** = 328)	Cases (***n*** = 290)	OR (95% CI)	OR (95% CI)
**Preeclampsia/eclampsia/toxemia**								
No	949 (98.3)	840 (98.4)	1.00	1.00	200 (98.5)	174 (95.6)	1.00	1.00
Yes	16 (1.7)	14 (1.6)	1.23 (0.48–3.16)^¥^	1.41 (0.50–3.97)^€^	3 (1.5)	8 (4.4)	ǂ	ǂ
**Gestational hypertension**^**§**^								
No	913 (97.5)	794 (98.0)	1.00	1.00	196 (97.5)	161 (97.0)	1.00	1.00
Yes	23 (2.5)	16 (2.0)	0.75 (0.35–1.60)^¥^	0.87 (0.38–1.97)^€^	5 (2.5)	5 (3.0)	1.09 (0.31–3.79)^¥^	1.37 (0.36–5.19)^€^
**Any hypertensive disorder**								
No	944 (96.0)	843 (95.6)	1.00	1.00	200 (96.2)	173 (93.0)	1.00	1.00
Yes	39 (4.0)	30 (3.4)	0.86 (0.46–1.59)^¥^	0.98 (0.50–1.90)^€^	8 (3.9)	13 (7.0)	2.83 (0.85–9.39)^¥^	5.61 (1.19–26.4)^€^
**Maternal smoking**								
No	757 (70.4)	665 (70.0)	1.00		166 (71.9)	145 (70.0)	1.00	
Yes	319 (29.6)	285 (30.0)	1.18 (0.77–1.82)*		65 (28.1)	62 (30.0)	1.23 (0.54–2.80)*	
**Low birth weight**								
Birth weight > 5.5 pounds	992 (93.5)	867 (92.3)	1.00	1.00	218 (91.9)	199 (91.7)	1.00	1.00
Birth weight ≤ 5.5 pounds	69 (6.5)	72 (7.7)	1.23 (0.82–1.85)	1.22 (0.76–1.96)^Ŧ^	19 (8.0)	18 (8.3)	0.95 (0.46–1.98)	0.85 (0.35–2.05)^Ŧ^
**High birth weight**								
Birth weight < 8.8 pounds	712 (92.6)	614 (90.2)	1.00	1.00	162 (92.1)	150 (91.5)	1.00	1.00
Birth weight ≥ 8.8 pounds	57 (7.4)	67 (9.8)	1.68 (1.07–2.62)	1.53 (0.95–2.48)^Ŧ^	14 (7.9)	14 (8.5)	1.34 (0.55–3.28)	1.35 (0.48–3.80)^Ŧ^
**Breastfed**								
No	640 (60.2)	582 (61.8)	1.00		130 (57.5)	134 (65.7)	1.00	
Yes	424 (39.9)	360 (38.2)	0.83 (0.61–1.13)		96 (42.5)	70 (34.3)	0.53 (0.29–0.99)	
**Soy-fed (formula made from soy)**								
No	827 (96.3)	722 (95.5)	1.00		181 (94.3)	163 (95.3)	1.00	
Yes	32 (3.7)	34 (4.5)	1.19 (0.70–2.01)		11 (5.7)	8 (4.7)	0.70 (0.27–1.84)	
**DES exposure during pregnancy**								
No	860 (96.9)	770 (97.2)	1.00		171 (98.3)	152 (98.1)	1.00	
Yes	28 (3.2)	22 (2.8)	0.85 (0.44–1.65)		3 (1.7)	3 (1.9)	1.00 (0.14–7.10)	
**Short gestational length at birth**								
Born ≥ 38 weeks	460 (87.3)	400 (84.8)	1.00	1.00	93 (91.2)	82 (85.4)	1.00	1.00
Born < 38 weeks	67 (12.7)	72 (15.2)	1.25 (0.82–1.92)	1.19 (0.76–1.85)^Ŧ^	9 (8.8)	14 (48.6)	2.39 (0.73–7.79)	2.11 (0.52–8.59)^Ŧ^

^¥^Adjusted for whether or not the participant was their mother’s first baby and for maternal smoking in that pregnancy

§Without preeclampsia/eclampsia

*Adjusted by birth order

^ǂ^Not enough participants in this category to generate an estimate

^€^Adjusted for whether or not the participant was their mother’s first baby, for maternal smoking in that pregnancy and excluding women who themselves had a preeclamptic pregnancy

^Ŧ^Adjusted for mother’s preeclampsia and smoking during the participant’s gestation

## Discussion

In the Two Sister Study, we observed that maternal preeclampsia and high birth weight showed some evidence of a positive association with young-onset breast cancer. We observed a weak inverse association between having been breastfed during infancy and young-onset breast cancer. Additionally, women born from pregnancies with preeclampsia or gestational hypertension who did develop young-onset breast cancer appeared to be at increased odds for the difficult-to-treat ER-negative subtype. Restricting attention to invasive breast cancer strengthened those associations.

Among the studies that have assessed the relationship between birth weight and young-onset breast cancer [12, 14, 16–23, 33], a positive association with birth weight was detected in the majority of them [14, 16–23]. Michels and Xue conducted a systematic review of some studies published in this area and noted a positive association between birth weight and risk of breast cancer [15]. Of the articles included that had evaluated only young-onset breast cancer, they reported that, overall, high birth weight appeared to be associated with increased risk (OR 1.25; 95% CI 1.14–1.38). Three of the studies [14, 16, 17] included in their meta-analysis reported a J-shaped or a U-shaped association between birth weight and breast cancer. The results of our study, which is based on women with breast cancer diagnosed before the age of 50, align with those previous results.

It has been proposed that the biological mechanism that underlies this birth weight association encompasses a complex interplay between the influences of growth factors and steroid hormones on mammary cells and epigenetic changes [15]. Maternal circulating hormones, such as estradiol and estriol, have been positively associated with birth weight in previous studies [34–36]. Certain metabolic byproducts of estrogen may be mutagenic [37] and may promote cell proliferation and cell differentiation [38]. Also, the levels of estriol in the umbilical cord have been reported to be positively correlated with birth weight [36]. Thus, the intrauterine environment of high birth weight babies may alter the normal mammary cell growth and impact later breast cancer risk. Other mechanisms that may explain the observed association between high birth weight and breast cancer involve growth factor pathways [39]. Cord blood concentrations of insulin-like growth factor-1 (IGF-1) have been reported to be positively associated with birth weight [40, 41]. In addition, IGF-1 is thought to be an important factor in fetal growth [42] and to be a potent mitogen of the mammary gland [43]. Based on their critical role in fetal growth and in the development of the mammary gland, IGF-1 and estrogen may play a role in the initiation and promotion of breast tumors.

Gestational length has been studied in association with risk of breast cancer [9, 44]. Maternal hormone levels consistently increase with gestational age [35]. Gestational length consequently impacts the fetus’ cumulative exposure in utero to maternal endogenous hormones, which could influence their breast cancer risk. Evidence from two meta-analyses reported no association between gestational length and breast cancer risk [9, 44], while results from the individual studies included in these articles provide heterogeneous evidence of both an increased [27] and decreased [19] risk of breast cancer associated with shorter gestational length. We did not observe a statistically significant association between gestational length and early-onset breast cancer. Our results are thus consistent with the previous meta-analyses [9, 44].

Inconsistent results have been reported in previous studies assessing the association between pre-eclampsia and daughter’s breast cancer risk [9, 12, 14]. Two studies with a small number of exposed participants reported a non-significant positive association, but the limited sample sizes of these studies limited their statistical power [10, 12]. Our study also found a positive association with breast cancer. This result contrasts with results from two record linkage studies [11, 27] from Sweden that reported a reduction in breast cancer risk among women whose mothers had pre-eclampsia or eclampsia during the participant’s gestation. These studies included both women with pre- and post- menopausal breast cancer. Additionally, the populations being analyzed overlapped partially. The discrepant results observed in our study could be due in part to the different age at breast cancer diagnosis in our study compared to both studies conducted by Ebkom and colleagues [11, 27] as young-onset and late-onset breast cancer can have different etiologies [45, 46]. One other consideration is that some women born to a preeclamptic pregnancy may have inherited a tendency to develop that complication in their own pregnancies and that particular reproductive history has been associated with reduced risk of breast cancer [31], reducing any increase that might have been due to the prenatal exposure. When we excluded women who themselves had a preeclamptic pregnancy, the association seen in our data was somewhat strengthened.

The results from our study are subject to some limitations. First, exposures were self-reported, which could have led to misclassification of the exposures. Also, limited numbers with the exposure prevented us from assessing gestational diabetes as a potential exposure.

Our results are strengthened by our study design, and a large number of young-onset breast cancer cases contributing to a case-only analysis. We had sufficient numbers to evaluate the association of perinatal factors with ER status among women affected by young-onset breast cancer. Finally, the sister-matched design helped us control for unmeasured confounding.

## Conclusion

In summary, in the present study, we observed small and imprecisely estimated positive associations between two prenatal exposures (high birth weight and being gestated in a preeclamptic pregnancy) and young-onset breast cancer. These results provide indirect support for the hypothesis that prenatal exposures affect the subsequent risk of breast cancer in young women. In addition, our results suggest that being born to a preeclamptic pregnancy may increase risk for young-onset breast cancer, especially for the ER-negative subtype. Future epidemiological research on the connection between maternal hypertensive disorders, birth weight, and young-onset breast cancer is warranted to help us gain a better understanding of the etiology of the disease.

## Supplementary information

**Additional file 1.**

## Data Availability

Collaborations based on data for the Two Sister Study are encouraged. See https://sisterstudy.niehs.nih.gov/english/twosisterstudy.htm

## Abbreviations

CI: Confidence interval
ER: Estrogen receptor
OR: Odds ratio
US: United States
RR: Risk ratio
BMI: Body mass index
IGF: Insulin-like growth factor
