# Supplementary material for: Perinatal and postnatal exposures and risk of young-onset breast cancer
Source: Breast Cancer Res. 2020 Aug 13;22:88. doi: 10.1186/s13058-020-01317-3 (PMC7427289; doi:10.1186/s13058-020-01317-3)
Supplement: Supplementary file 1 — Additional file 1. [file 13058_2020_1317_MOESM1_ESM.docx]

| Table 6: Breast cancer odds ratio (ORs) in relation to participant's in-utero exposures by ER status, with counts including only participants with complete information on exposure of interest. | | | | | | | |  |  |  |  |  |
| --- | --- | --- | --- | --- | --- | --- | --- | --- | --- | --- | --- | --- |
|  | **ER + cases (1161 case subjects, 1321 control subjects)** | | | | **ER - cases (290 case subjects, 328 control subjects)** | | | | | | | |
| **Characteristics** | **Controls (n=1321)** | **Cases (n=1161)** | **OR (95 % CI)** | **OR (95 % CI)** | **Controls (n=328)** | **Cases (n=290)** | **OR (95 % CI)** | **OR (95 % CI)** | | | | |
| **Preeclampsia/Eclampsia**  **/Toxemia** |  |  |  |  |  |  |  |  | | | | |
| No | 949 (98.3) | 840 (98.4) | 1.00 | 1.00 | 200 (98.5) | 174 (95.6) | 1.00 | 1.00 | | | | |
| Yes | 16 (1.7) | 14 (1.6) | 1.23 (0.48-3.16)^¥^ | 1.41 (0.50-3.97)^€^ | 3 (1.5) | 8 (4.4) | ǂ | ǂ | | | | |
| **Gestational Hypertension^§^** |  |  |  |  |  |  |  |  | | | | |
| No | 913 (97.5) | 794 (98.0) | 1.00 | 1.00 | 196 (97.5) | 161 (97.0) | 1.00 | 1.00 | | | | |
| Yes | 23 (2.5) | 16 (2.0) | 0.75 (0.35-1.60)^¥^ | 0.87 (0.38-1.97)^€^ | 5 (2.5) | 5 (3.0) | 1.09 (0.31-3.79)^¥^ | 1.37 (0.36-5.19)^€^ | | | | |
| **Any hypertensive disorder** |  |  |  |  |  |  |  |  | | | | |
| No | 944 (96.0) | 843 (95.6) | 1.00 | 1.00 | 200 (96.2) | 173 (93.0) | 1.00 | 1.00 | | | | |
| Yes | 39 (4.0) | 30 (3.4) | 0.86 (0.46-1.59)^¥^ | 0.98 (0.50-1.90)^€^ | 8 (3.9) | 13 (7.0) | 2.83 (0.85-9.39)^¥^ | 5.61 (1.19-26.4)^€^ | | | | |
| **Maternal smoking** |  |  |  |  |  |  |  |  | | | | |
| No | 757 (70.4) | 665 (70.0) | 1.00 |  | 166 (71.9) | 145 (70.0) | 1.00 |  | | | | |
| Yes | 319 (29.6) | 285 (30.0) | 1.18 (0.77-1.82)^*^ |  | 65 (28.1) | 62 (30.0) | 1.23 (0.54-2.80)^*^ |  | | | | |
| **Low birthweight** |  |  |  |  |  |  |  |  | | | | |
| Birthweight > 5.5 pounds | 992 (93.5) | 867 (92.3) | 1.00 | 1.00 | 218 (91.9) | 199 (91.7) | 1.00 | 1.00 | | | | |
| Birthweight ≤ 5.5 pounds | 69 (6.5) | 72 (7.7) | 1.23 (0.82-1.85) | 1.22 (0.76-1.96)^Ŧ^ | 19 (8.0) | 18 (8.3) | 0.95 (0.46-1.98) | 0.85 (0.35-2.05)^Ŧ^ | | | | |
| **High Birthweight** |  |  |  |  |  |  |  |  | | | | |
| Birthweight < 8.8 pounds | 712 (92.6) | 614 (90.2) | 1.00 | 1.00 | 162 (92.1) | 150 (91.5) | 1.00 | 1.00 | | | | |
| Birthweight ≥ 8.8 pounds | 57 (7.4) | 67 (9.8) | 1.68 (1.07-2.62) | 1.53 (0.95-2.48)^Ŧ^ | 14 (7.9) | 14 (8.5) | 1.34 (0.55-3.28) | 1.35 (0.48-3.80)^Ŧ^ | | | | |
| **Breast-fed** |  |  |  |  |  |  |  |  | | | | |
| No | 640 (60.2) | 582 (61.8) | 1.00 |  | 130 (57.5) | 134 (65.7) | 1.00 |  | | | | |
| Yes | 424 (39.9) | 360 (38.2) | 0.83 (0.61-1.13) |  | 96 (42.5) | 70 (34.3) | 0.53 (0.29-0.99) |  | | | | |
| **Soy-fed (formula made from soy)** |  |  |  |  |  |  |  |  | | | | |
| No | 827 (96.3) | 722 (95.5) | 1.00 |  | 181 (94.3) | 163 (95.3) | 1.00 |  | | | | |
| Yes | 32 (3.7) | 34 (4.5) | 1.19 (0.70-2.01) |  | 11 (5.7) | 8 (4.7) | 0.70 (0.27-1.84) |  | | | | |
| **DES exposure during pregnancy** |  |  |  |  |  |  |  |  | | | | |
| No | 860 (96.9) | 770 (97.2) | 1.00 |  | 171 (98.3) | 152 (98.1) | 1.00 |  | | | | |
| Yes | 28 (3.2) | 22 (2.8) | 0.85 (0.44-1.65) |  | 3 (1.7) | 3 (1.9) | 1.00 (0.14-7.10) |  | | | | |
| **Short gestational length at birth** |  |  |  |  |  |  |  |  | | | | |
| Born ≥ 38 weeks | 460 (87.3) | 400 (84.8) | 1.00 | 1.00 | 93 (91.2) | 82 (85.4) | 1.00 | 1.00 | | | | |
| Born < 38 weeks | 67 (12.7) | 72 (15.2) | 1.25 (0.82-1.92) | 1.19 (0.76-1.85)^Ŧ^ | 9 (8.8) | 14 (48.6) | 2.39 (0.73-7.79) | 2.11 (0.52-8.59)^Ŧ^ | | | | |
| ¥Adjusted for whether or not the participant was their mother's first baby and for maternal smoking in that pregnancy | | | | |  | |  | | |  | |  |
| § Without preeclampsia/eclampsia |  |  |  |  |  |  |  |  | | | | |
| *Adjusted by birth order |  |  |  |  |  |  |  |  | | | | |
| ǂ Not enough participants in this category to generate an estimate | | |  |  |  | |  | |  | |  | |
| ^€^ Adjusted for whether or not the participant was their mother's first baby, for maternal smoking in that pregnancy and excluding women who themselves had a preeclamptic pregnancy | | | | | | | |  |  |  |  |  |
| ^Ŧ^Adjusted for mother's preeclampsia and smoking during the participant's gestation. | | | |  |  | |  | | |  | |  |
